# Socioeconomic and Geographic Differences in Mammography Trends Following the 2009 USPSTF Policy Update

**DOI:** 10.1001/jamanetworkopen.2024.58141

**Published:** 2025-02-05

**Authors:** Jason Semprini, Loren Saulsberry, Olufunmilayo I. Olopade

**Affiliations:** 1 Department of Public Health, Des Moines University College of Health Sciences, West Des Moines, Iowa; 2 Department of Public Health Sciences, The University of Chicago, Chicago, Illinois; 3 Section of Hematology/Oncology, Department of Medicine, University of Chicago Medicine Comprehensive Cancer Center, Chicago, Illinois

## Abstract

**Importance:**

In 2024, the US Preventive Services Task Force (USPSTF) reversed a 2009 policy recommending only females aged 50 to 74 years complete a biennial mammogram. Understanding whether females facing heterogeneous breast cancer risks responded to the 2009 guidance may illuminate how they may respond to the latest policy update.

**Objective:**

To evaluate whether the 2009 policy was associated with changes in mammography screening in females no longer recommended to complete a biennial mammogram and whether these changes varied by factors associated with breast cancer risk.

**Design, Setting, and Participants:**

The difference-in-differences design compared biennial mammogram trends in the exposed groups (aged 40-49 and ≥75 years) with trends of the unexposed groups (aged 50-64 and 65-74 years), before and after the 2009 update. Population-based, repeated cross-sectional survey data came from the Behavioral Risk Factor Surveillance System (BRFSS) biennial cancer screening module (2000-2018). The sample was restricted to females between ages 40 and 84 years. Data were analyzed from March 1 to June 30, 2024.

**Main Outcomes and Measures:**

The outcome was a binary variable indicating whether the respondent reported a mammogram in the past 2 years (biennial). After 2009, females aged 40 to 49 and 75 or older years were exposed to the policy update, as a complete biennial mammogram was recommended. Subgroup analyses included race and ethnicity, educational level, household income, smoking history, current binge drinking status, and state of residence.

**Results:**

The sample included 1 594 834 females; 75% reported a biennial mammogram. In those aged 40 to 49 years, the USPSTF update was associated with a 1.1 percentage-point (95% CI, −1.8% to −0.3 percentage points) decrease in the probability of a biennial mammogram, with the largest decreases in the non-Hispanic Black population (−3.0 percentage points; 95% CI, −5.5% to −0.5 percentage points). In the aged 75 years or older group, the USPSTF update was associated with a 4.8 percentage-point decrease (95% CI, −6.3% to −3.5 percentage points) in the probability of a biennial mammogram, with significant heterogeneity by race and ethnicity, binge drinking status, and state residence.

**Conclusions and Relevance:**

In this study, socioeconomic factors were associated with differences in how females responded to the 2009 USPSTF mammography recommendation. Whether the 2024 update considered such differences is unclear. These findings suggest that including risk assessment into future USPSTF policy updates may improve adoption of risk-reducing interventions and shorten the time to diagnosis and treatment for high-risk patients.

## Key Points

**Question:**

Did female biennial mammography screening rates change after the US Preventive Services Task Force (USPSTF) 2009 policy update?

**Findings:**

This cross-sectional study of 1.6 million females found reduced mammography rates after the USPSTF 2009 update for those no longer recommended to complete a biennial mammogram. These decreases varied by age, race and ethnicity, binge drinking status, and state of residence.

**Meaning:**

The findings of this study suggest that patient-level variables related to breast cancer risk are associated with differences in how women responded to the 2009 USPSTF mammography guidance.

## Teaser

This cross-sectional study examines changes in the levels of mammography screening after the 2009 US Preventive Services Task Force (USPSTF) guidance regarding the recommended age range for biennial mammograms.

## Introduction

Although disparities persist, breast cancer mortality in the US has been decreasing for decades.^1^ This decrease has been attributed to earlier detection and improved therapies.^2^ The empirical evidence, however, suggests that the association between mammograms, the reference standard for breast cancer screening, includes a tradeoff that balances the benefits of reducing advanced-stage cancers with the harms of identifying potentially nonfatal tumors.^3^ Confronted by this cost-benefit assessment, clinicians and patients have relied on the recommendations from the US Preventive Services Task Force (USPSTF). The USPSTF is an independent panel of experts who synthesize evidence to provide guidance on who should and should not complete specific health care services. Policymakers also rely on the USPSTF guidance, as federal law requires insurance providers to fully cover preventive services recommended by the USPSTF.^4^ Prior to 2009, the USPSTF recommended that all females older than 40 years complete a mammogram every 2 years.^5^ More than 70% of American females adhered to this recommendation.^6^

In December 2009, the USPSTF updated their mammogram policy to recommend that, beginning in 2010, females between ages 50 and 74 years complete a biennial mammogram.^7,8^ This updated guidance further recommended that screening in females aged 40 to 49 years be considered on a case-by-case basis.^9^ For females older than 75 years, the 2009 update stated that the “evidence is insufficient to assess the balance of benefits and harms of screening mammography.”^9^ This policy update was met with confusion by patients^10^ and controversy from clinicians, who continued recommending biennial mammograms given the conflicting guidance from the USPSTF and other public health advocacy groups.^11,12,13^ After 15 years, the USPSTF has reversed their 2009 mammography recommendation,^14,15^ issuing guidance extending the biennial mammography recommendation to females aged 40 to 49 years.^16^

The USPSTF updated their mammography recommendation to align with current evidence and maximize potential benefit from early detection while minimizing potential harms in populations with limited expected benefit from screening.^17,18^ However, to maximize benefits of earlier detection and minimize harms from overdiagnosis and higher health care costs, the guidance must change behavior. There is limited evidence that patients actually change their behavior following updated USPSTF guidance^19,20,21,22,23^ and the evidence specific to the USPSTF mammography guidance has been mixed.^24,25,26,27,28^ One problem within the existing literature stemmed from the analysis of claims-based data or convenience sample cohorts from which findings cannot be generalized to subpopulations outside the system.^29,30,31^ Additionally, studies on the existing evidence examining mammography trends have focused on short-term changes and have not attempted to account for unobserved factors unrelated to the USPSTF policy update that potentially impacted mammography screening.^32,33^ Moreover, existing population-level research often modeled trends of females who were not affected by the policy update (aged 50-74 years) as a single group, ignoring considerable differences in health care access and breast cancer risk within this broad age range. A more fundamental problem with the existing evidence (ie, evidence that has informed the latest USPSTF mammography policy update) is the limited attention to individual risk heterogeneity.^4^ Failing to understand how different subpopulations change their mammography trends in response to new policy guidance could have major implications for cancer disparities.

Despite the reversal of the most recent USPSTF mammography guideline, controversy remains.^34,35,36,37^ To inform the ongoing debate and future updates, our study aimed to evaluate whether the 2009 USPSTF policy update impacted biennial mammogram trends. Prior evidence had limited generalizability and typically relied on research designs that may not have accounted for dynamic mammography trends or selection bias, but the more pressing matter was the lack of attention to breast cancer risk heterogeneity. Until we understand how populations at different risks of adverse breast cancer outcomes differentially change their behavior in light of new preventive care guidance, policymakers have limited ability to design effective public health guidance. Our goal is to contribute such evidence and inform efforts to leverage public health guidance as a tool for advancing health equity.

## Methods

### Data and Sample

We retrieved publicly available, repeated cross-sectional data from the Centers for Disease Control and Prevention Behavioral Risk Factor Surveillance System (BRFSS).^38^ The BRFSS is a population-based, nationally representative survey administered by phone and has served as the reference standard survey instrument for assessing public health trends.^39^ We specifically analyzed BRFSS data from the biennial breast and cervical cancer screening module, which asks female respondents whether they have ever had a mammogram and, if so, how long it has been since the most recent mammogram.^38,40^ Missing responses, either because the respondent did not know or refused to answer, were excluded (<4% of the weighted sample). Because all data were obtained from publicly available, secondary deidentified data, this study did not constitute human research and does not require institutional review board review or exemption according to the Common Rule (45 CFR §46). This study adhered to the Strengthening the Reporting of Observational Studies in Epidemiology (STROBE) reporting guideline for cross-sectional studies.^41^

The BRFSS breast cancer screening module included even years from 2000 to 2022. We excluded 2020 and 2022 to avoid issues related to the COVID-19 pandemic.^42^ In addition to restricting our analyses to females, we restricted the analysis to those between the ages of 40 and 84 years, as those age groups were recommended to complete a biennial mammogram prior to the 2009 USPSTF policy update.^5,8,9^ Respondents from all 50 states and the District of Columbia were included in the sample.

### Variables and Exposure

Our outcome of interest was derived from the 2 mammography BRFSS questions and measured as a binary variable indicating whether the respondent had received a mammogram in the past 2 years. To account for differences in mammography screening rates, all analyses were adjusted for age, race and ethnicity, employment status, educational level, household income, and marital status.

We also used these independent variables to conduct a set of subgroup analyses by race and ethnicity (Hispanic, non-Hispanic Black, and non-Hispanic White), educational level (college vs no college), and annual household income (<$75 000 or≥$75 000). Race and ethnicity was identified by self-report during the BRFSS interview. There were more responses to the self-reported race and ethnicity question in BRFSS; however, due to limited sample and our research question, we did not conduct subgroup analyses with any other racial and ethnic category. Our secondary set of subgroup tests were derived from a BRFSS-computed smoking status variable, which we dichotomized into any history of smoking vs no history of smoking, and a BRFSS-computed binary variable indicating the respondent’s self-reported current binge drinking behavior.^43,44^ Given the importance of access and policy for influencing mammography trends, and heterogeneous rates of increasing breast cancer incidence by state, we conducted another analysis within each state.^45,46,47^

The exposure of interest was the December 2009 policy update.^9^ We considered the policy to take effect in 2010 and created a binary exposure variable based on the respondent’s age group. There were 2 age groups exposed to the policy update: females aged 40 to 49 years and those aged 75 years or older. Females aged 50 to 74 years were not exposed to the policy update, as their recommendation did not change. To increase the validity of our design, we evaluated the 2 exposed groups separately and with comparable age groups. We compared the trends in females aged 40 to 49 years with the trends in those aged 50 to 64 years (pre-Medicare sample). We compared the trends in females aged 75 years or older with the trends in those aged 65 to 74 years (Medicare sample).

### Statistical Analysis

To evaluate the 2009 USPSTF mammogram policy update, we constructed a difference-in-differences design.^48^ Commonly used in health policy evaluations, difference-in-differences designs account for unobserved baseline differences between exposed and unexposed groups, as well as temporal trends consistent across all groups.^48^ Herein, our difference-in-differences design compared the biennial mammogram trends in the exposed group with the trends in the unexposed group before and after the 2009 policy update. We excluded 2010 as a washout period. To avoid confounding from differences in health care use and access between the age groups, the 2 exposed groups were analyzed separately (aged 40-49 and 50-64 years; and 65-74 and ≥75 years).

Each analysis was constructed as a linear probability regression model,^49^ which estimates the change in the probability of reporting a biennial mammogram. All models were adjusted by BRFSS-supplied probability sampling weights.^50^ For inference, we report 95% CIs based on estimated SEs robust to heteroskedasticity and clustered at the state level.^51^ In addition to adjusting for sociodemographic control variables, our regression included year to account for temporal trends, state to account for state-level differences, and year × state interaction fixed effects to account for state policy changes or events. Following common practice, we tested our model’s validity with an event-history study.^52,53,54,55^ With 2-sided testing, the significance threshold was set at α = .05. The eAppendix in Supplement 1 provides a detailed description of the regression model. Data were analyzed from March 1 to June 30, 2024. Statistical analysis was conducted with Stata, version 18 (StataCorp LLC).

## Results

**Figure 1.**
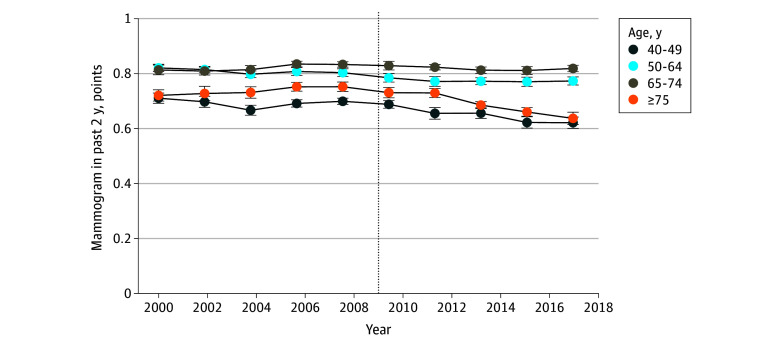
Unadjusted Population-Weighted Trends in the Proportion of Females Reporting a Completed Mammogram in the Past 2 Years The vertical line at year 2009 represents the year in which the US Preventive Services Task Force updated their mammogram policy from recommending a biannual mammogram for females aged 40 years and older to recommending a biannual mammogram for those aged 50 to 74 years. Error bars indicate 95% CI of the mean. The proportions are on a binary (0-1) scale.

Our sample included 1 594 834 respondents. Before the 2009 policy update, 75% of the sample reported completing a mammogram in the past 2 years. Table 1 presents the sample composition and baseline (2000-2009) biennial mammogram rates for the full sample and by individual characteristics. Even before the 2009 update, the age groups unexposed to the policy update (age, 50-74 years) reported higher (81%-82%) rates of biennial mammograms than those aged 40 to 49 years (69%) and 75 years or older (74%). Among subgroups, females reporting $75 000 or more in annual household income reported the highest rate of biennial mammograms (82.8%), whereas those who reported the health behavior of binge drinking in the past month reported the lowest rate (71.1%). Figure 1 shows the trends in biennial mammograms for females aged 40 to 49, 50 to 64, 65 to 74, and 75 years or older.

**Table 1.** Summary Statistics

Variable	Sample, No. (%)	Proportion receiving biennial mammogram (2000-2009), mean % (SD)
Age range, y		
40-49	462 502 (29)	69.3 (46.1)
50-64	615 606 (39)	80.8 (39.4)
65-74	279 096 (18)	82.0 (38.4)
75-84	237 630 (15)	73.7 (44.0)
Race and ethnicity		
Hispanic	154 380 (10)	73.1 (44.3)
Non-Hispanic Black	169 052 (11)	77.7 (41.6)
Non-Hispanic White	1 167 418 (73)	76.6 (42.3)
Employment status		
Not employed	950 521 (60)	75.5 (43.0)
Employed	644 313 (40)	77.3 (41.9)
Educational level		
Not college educated	693 367 (43)	72.7 (44.6)
College educated	901 467 (57)	79.2 (40.1)
Annual household income		
Low-income household (<$75 000)	1 170 608 (73)	74.0 (43.9)
High-income household (≥$75 000)	424 226 (27)	82.8 (37.8)
Marital status		
Not married	658 666 (41)	72.2 (44.8)
Married	936 168 (59)	78.9 (40.1)
Smoking status		
No history of smoking	923 409 (58)	78.2 (41.3)
History of smoking	671 425 (42)	73.7 (44.0)
Reported binge drinking		
No recent binge drinking	1 358 799 (85)	80.1 (40.0)
Reported binge drinking in past month	236 035 (15)	71.1 (45.3)
Total sample	1 594 834 (100)	76.2 (42.3)

**Figure 2.**
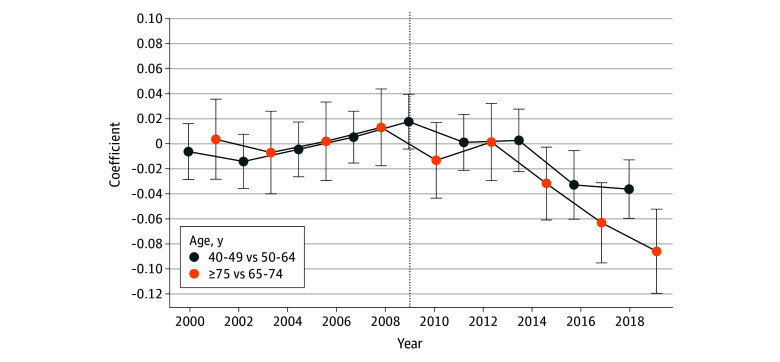
Event History Linear Probability Model, Testing the Parallel Trends Assumption of the Difference-in-Differences Design Each coefficient and 95% CI compares the relative difference between exposed and unexposed age groups in the changing biannual mammogram trends from the baseline year 2000. The vertical line at year 2009 represents the year in which the US Preventive Services Task Force updated their mammogram policy from recommending a biannual mammogram for females aged 40 years or older to recommending a biannual mammogram for females aged 50 to 74 years. Error bars indicate 95% CIs of the estimated association. The proportions are on a binary (0-1) scale.

Following the USPSTF policy update, biennial mammography rates for females aged 40 to 49 years declined to 68.2% (95% CI, 67.5%-69.0%) and for females aged 75 years or older declined to 68.9% (95% CI, 67.4%-70.2%). For females aged 40 to 49 years, the USPSTF policy update was associated with a 1.1 percentage point decrease in the probability of reporting a biennial mammogram (95% CI, −1.8% to −0.3 percentage points) (Table 2). This estimate represents a relative 1.6% drop from baseline screening rates (<2010 biennial mammogram rate, 69.3%). For females aged 75 years or older, the USPSTF policy update was associated with a 4.8 percentage-point decrease in the probability of reporting a biennial mammogram (95% CI, −6.3% to −3.5 percentage points) (Table 2), which represents a relative 6.3% decrease from baseline screening rates (<2010 biennial mammography rate, 73.7%). In the age groups 40 to 49 years and 75 years and older females, the results of our event-history analysis found no significant differences in biennial mammography trends between exposed and unexposed age groups before 2010 (Figure 2).

**Table 2.** Estimated Association Between 2009 USPSTF Policy Update and Probability of Reporting a Biennial Mammogram^a^

Variable	Coefficient (95% CI)	
	Age 40-49 y	Age ≥75 y
Overall	−1.1 (−1.8 to −0.3)^b^	−4.8 (−6.3 to −3.5)^c^
Race and ethnicity		
Hispanic	−0.7 (−3.4 to 2.0)	−6.2 (−11.7 to −0.7)^b^
Non-Hispanic Black	−3.0 (−5.5 to −0.5)^b^	−1.0 (−3.5 to 1.5)
Non-Hispanic White	−0.7 (−1.7 to 0.3)	−4.7 (−5.9 to −3.5)^b^
Educational level		
No college degree	−0.9 (−2.1 to 0.3)	−4.7 (−6.1 to −3.3)^c^
College degree	−1.3 (−2.1 to −0.5)^d^	−5.4 (−7.0 to −3.8)^c^
Annual household income		
Low-income	−0.9 (−1.9 to 0.1)	−4.8 (−6.2 to −3.4)^c^
High-income	−1.8 (−3.0 to −0.6)^d^	−5.2 (−7.8 to −2.7)^c^
Smoking history		
Never smoker	−1.5 (−2.3 to −0.7)^c^	−4.9 (−6.5 to −3.3)^c^
History of smoking	−1.2 (−2.4 to 0.0)	−4.6 (−6.2 to −3.0)^c^
Reported binge drinking		
No recent binge drinking	−1.1 (−2.3 to 0.1)	−4.8 (−6.8 to −2.8)^c^
Binge drinking in past month	−1.2 (−3.9 to 1.5)	−0.8 (−6.5 to 4.9)

Abbreviation: USPSTF, US Preventive Services Task Force.

^a^ The results from the difference-in-differences, linear probability regression model. Each coefficient estimates the association between the USPSTF policy recommendation update on the probability of reporting a biennial mammogram. Inference is based on SEs robust to heteroskedasticity. Each age and subgroup was estimated separately.

^b^ *P* < .05.

^c^ *P* < .001.

^d^ *P* < .01.

**Figure 3.**
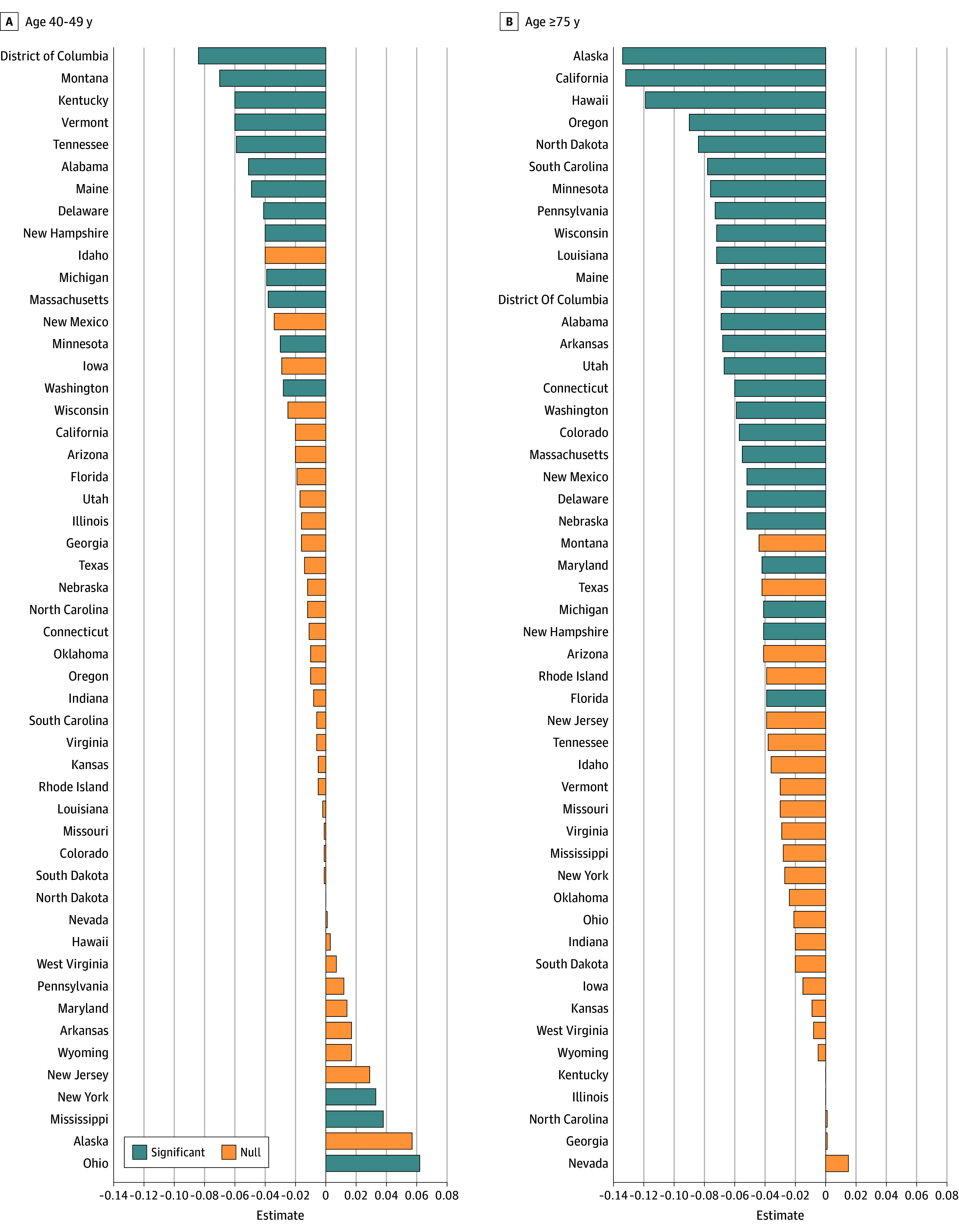
Each State’s Coefficient Estimating the Association Between the 2009 US Preventive Services Task Force Policy Update and Biannual Mammograms Difference in estimation between policy update and biennial mammograms in females aged 40 to 49 years (A) vs those aged 75 years or older (B). States are ordered by the magnitude of the estimate and color coded by the statistical significance of the estimate (*P* < .05).

Within the 40- to 49-year age group, the largest estimated association between the policy update and biennial mammograms was found in the non-Hispanic Black population (estimate = −3.0 percentage points; 95% CI, −5.5% to −0.5 percentage points). However, based on the 95% CIs, the estimated association between the USPSTF policy update and biennial mammograms was consistent between racial and ethnic subgroups, educational levels, household income, smoking history, and binge drinking status (Table 2). When examining differences by state, we observed that 12 states (Alabama, Delaware, Kentucky, Maine, Massachusetts, Michigan, Minnesota, Montana, New Hampshire, Tennessee, Vermont, and Washington) and the District of Columbia were found to have a statistically significant negative association between the policy update and biennial mammograms (Figure 3A). The largest association was found in the District of Columbia, where the USPSTF policy update was associated with an 8.4 percentage-point reduction in the probability of reporting a biennial mammogram.

Similarly, within the aged 75 years or older age group, we did not observe any meaningful differences between the policy update and biennial mammograms by educational level, household income, or smoking status. While the estimated association between the policy update and biennial mammograms was higher in females who did not report the health behavior of binge drinking (estimate = −4.8 percentage points; 95% CI, −6.8% to −2.8 percentage points) compared with females who reported binge drinking (estimate = −0.8 percentage points; 95% CI, −6.5% to 4.9 percentage points), the imprecise estimates among females who reported binge drinking limits our inference to distinguish statistically different effects. The policy update’s association with biennial mammograms differed significantly by race and ethnicity. Not only are these differences by race and ethnicity clinically meaningful and statistically significant, they also contrast with the heterogeneity within the age 40- to 49-year group. For females aged 75 years or older, there was no association between the USPSTF update and biennial mammograms for non-Hispanic Black females (estimate = −0.1 percentage points; 95% CI, −3.5% to 1.5 percentage points). Conversely, we estimated a statistically significant decrease for Hispanic females estimate = −6.2 percentage points; 95% CI, −11.7% to −0.7 percentage points) and non-Hispanic White females (estimate = 4.7 percentage points; 95% CI, −5.9% to −3.5 percentage points). When examining differences by state, we observed that 25 states (Alabama, Alaska, Arkansas, California, Colorado, Connecticut, Delaware, District of Columbia, Florida, Hawaii, Louisiana, Maine, Maryland, Massachusetts, Michigan, Minnesota, Nebraska, New Hampshire, New Mexico, North Dakota, Oregon, Pennsylvania, South Carolina, Utah, Washington, and Wisconsin) and the District of Columbia were found to have a statistically significant negative association between the policy update and biennial mammograms (Figure 3B). For females aged 75 years or older in 3 states (Alaska, California, and Hawaii), the association between the 2009 policy update and probability of biennial mammograms was a decrease of more than 10 points.

## Discussion

Since 2010, females with average breast cancer risk between the ages of 40 and 49 years and those older than 75 years have not been recommended by the USPSTF to receive a biennial mammogram. Analyzing a large population-based and nationally representative random sample of survey respondents, we presented evidence suggesting that this policy reduced the likelihood of completing a biennial mammogram in females exposed to the policy update compared with those not exposed. Overall, our estimates were consistent with the literature that found relatively small, but statistically significant, decreases in mammography rates after the USPSTF policy for females younger than 50 years.^24,25^ Contrary to other research,^24,25^ we observed that the largest reduction in biennial mammograms following the USPSTF policy was found in non-Hispanic Black females aged 40 to 49 years. Our results in the older age group (≥75 years) of non-Hispanic Black women were consistent with evidence regarding heterogeneity by race and ethnicity.^27,28^ We also examined other sociodemographic factors and behaviors associated with breast cancer risk.^43,56^ In the 75 years or older age group, in addition to observing variation by race and ethnicity, we noted a reduction in the biennial mammograms following the USPSTF update in females who did not binge drink, but no substantial change in those who reported binge drinking in the past month.^55^

Why might Black females aged 40 to 49 years have a different response to new mammogram guidance than Black females aged 75 years or older? And why did we observe a different response between the 2 age groups among Hispanic and White females? Diverse responses within each racial and ethnic group across age could be associated with differences in risk perception, medical history, and trust in medical authorities.^57,58,59,60^ Younger women may prioritize different risks and benefits, while older women often possess more health care experiences and awareness of breast cancer risks.^59,61,62^ Socioeconomic disparities and cultural influences could further mediate the behavioral response to public health guidance over each patient’s life course.^57,63^ Incorporation of appropriate measures of such health-related social influencers, such as the Social Vulnerability Metric, may lend insights for appropriate risk assessment across populations.^64^ Policymakers must recognize these complexities and leverage evidence-based tools to tailor guidance that accommodates patients’ experiences (ie, access to care, cultural/environmental contexts, and perspectives). Future research could further illuminate the key factors explaining racial and ethnic heterogeneity in response to USPSTF guidance.^56^ Specifically, understanding behavioral and social mechanisms explaining adherence and nonadherence to updated public health guidance could help policymakers and clinicians mitigate adverse outcomes. At present, the full influence of the USPSTF policy update on patient outcomes is unknown, let alone the potential disparate influence by race and ethnicity or social vulnerability across age groups on receipt of guideline-concordant mammography screening.

Biennial mammograms decreased in both age groups within 9 states following the 2009 policy update. Eight of these 9 states (Delaware, District of Columbia, Maine, Massachusetts, Michigan, Minnesota, New Hampshire, and Washington) expanded Medicaid under^65^ or before the Affordable Care Act,^66,67^ and are considered high-performing or innovative health care systems.^68,69,70^ The lone outlier was Alabama, which has not expanded Medicaid and does not rank high on similar health system metrics. As state health systems prepare for the latest USPSTF update, future research should investigate the economic, cultural, and environmental factors contributing to this state heterogeneity. Additionally, states where mammography patterns did not decrease after the 2009 USPSTF update (Illinois, Iowa, Kansas, Missouri, New Jersey, North Carolina, Rhode Island, and Virginia) also have some of the nation’s fastest rising incidence of female breast cancer,^68^ which warrants further investigation.

The USPSTF serves a critical function for patients and health care professionals, as early detection remains a pillar for breast cancer control.^71^ Advocates have approved of the latest USPSTF update as a tool for promoting equity.^35,36^ However, the potential harm from a policy’s unintended consequences must also be considered, especially in policies designed to promote equity.^72^ An individual patient’s risk of breast cancer is multifaceted.^73,74,75,76^ To maximize the potential benefits of early detection and minimize potential harms, ideally patients and clinicians base screening decisions from a comprehensive risk assessment.^77^ Unfortunately, most physician visits involve less than 5 minutes of conversation^78^; this is not nearly enough time to conduct a comprehensive breast cancer risk assessment. Instead, for low-risk individuals, patients and clinicians rely on USPSTF guidance, which is simplified by information easily gathered in a short office visit (eg, age). Population-level cancer screening policies also involve a complicated risk-benefit assessment, encompassing patient and societal perspectives. As risk stratification tools become more effective and less expensive, we might expect public health authorities to consider information more than age.^79^ One example is the current USPSTF guidance related to lung cancer screening, which recommends screening by age and smoking history.^80^ Clearly, more research investigating how to design and implement risk-stratified screening recommendations in diverse patient populations should be prioritized as in the Wisdom Study.^16^ In the absence of risk-stratified screening tools and recommendations, the burden of such an assessment will continue to be shouldered by the patient.^81,82^

### Limitations

This study has limitations. The BRFSS data are self-reported, potentially susceptible to recall bias. Respondents may misrepresent certain behaviors (ie, smoking, binge drinking), so these subgroup results should be interpreted with caution. Furthermore, many populations (ie, uninsured, sexual and gender minorities, rural adults, and indigenous ethnicity) were underrepresented in the BRFSS or did not have identifying variables through all years. Information on respondent medical history and community identifiers was also unavailable in the BRFSS. Regarding our design, internal validity relies on assumptions that can be tested, but not proven.

## Conclusions

In 2009, the USPSTF updated their mammogram policy from recommending biennial screening for all women older than 40 years to biennial screening for women aged 50 to 74 years. This policy update has since been reversed. The results of this cross-sectional study suggest that the 2024 mammography update may increase breast cancer screening rates differently across the population of females aged 40 to 49 years. Within each age group exposed to the 2009 policy update, we identified differences in the mammogram trends by age, race and ethnicity, health behaviors, and state of residence. When the USPSTF revisits their mammography recommendations, we encourage considering risk assessments in future guidance.

## Notes

Supplement 1. **eAppendix.** Technical Information

Supplement 2.
Data Sharing Statement
