# Supplementary material for: Socioeconomic and Geographic Differences in Mammography Trends Following the 2009 USPSTF Policy Update
Source: JAMA Netw Open. 2025 Feb 5;8(2):e2458141. doi: 10.1001/jamanetworkopen.2024.58141 (PMC11800017; doi:10.1001/jamanetworkopen.2024.58141)
Supplement: Supplement 1. — eAppendix. Technical Information [file jamanetwopen-e2458141-s001.pdf]

Supplementary Online Content

Semprini J, Saulsberry L, Olopade OI. Socioeconomic and geographic differences in mammography trends following the 2009 US Preventive Services Task Force policy update. *JAMA Netw Open*. 2024;8(2):e2458141.
doi:10.1001/jamanetworkopen.2024.58141

eAppendix. Technical Information

This supplementary material has been provided by the authors to give readers additional information about their work.

© 2025 Semprini J et al. *JAMA Network Open.*

eAppendix. Technical Information

To estimate the causal effect of the USPSTF 2009 mammogram policy update on bi-annual screening, we constructed a Difference-in-Differences design with state, year, and state-year interaction fixed effects. The outcome of interest is a binary variable indicating if the respondent reported a mammogram in the past year. This outcome can me modelled as the probability of receiving a bi-annual mammogram:

1) $Y_{iast} = P(RECEIVED\ MAMMOGRAM\ IN\ PAST\ 2 - YEARS|\ i = individual, a = age\ group, s = state,\ t = year)$

This binary outcome variable was derived from two BRFSS questions. The first question was 1) "The next questions are about breast and cervical cancer. Have you ever had a mammogram?" (including an interviewer note to indicate that "a mammogram is an x-ray of each breast to look for breast cancer") (BRFSS 2018 Questionnaire); the second question was 2) "How long has it been since you had your last mammogram?" This question included a prompt: within the past year, within the past two years (1 year ago but less than 2 years ago), within the past 3 years, etc. up until 5 or more years ago. Based on the responses from these two questions, a respondent's outcome variable Y_{iast} = 1 if the respondent reported ever having a mammogram AND having reported their most recent mammogram in the past 2 years, zero else.

We then construct a linear probability regression model:

2) $Y_{iast} = UPSTF_{at} + STATE_s + YEAR_t + STATE_s * YEAR_t + X'_{ist}$

Where $USPSTF_{at}$ indicates if the respondent was exposed to the policy update by their age-group = a (40-49 or 75+), in year t > 2010. The $STATE_s$ fixed-effect adjusts for state-level, time-invariant differences in screening rates. The $YEAR_t$ fixed-effect adjusts for global temporal trends in screening rates. The interaction between $STATE_s$ and $YEAR_s$ adjusts for state-level differences over time which affect each age-group similarly. The X'_{iast} vector includes a set of binary, exogenous control variables (five-year age groups, race/ethnicity, employment status, education level, household income, marital status). The initial post-exposure year, 2010, is excluded as a "wash-out" period.

Equation 2 is estimated via a linear probability regression model, with estimated standard errors robust to heteroskedasticity clustered at the state-level. Each exposed group is analyzed separately and repeated for the specified subgroups described in the manuscript.

Our design estimates an unbiased effect of the USPSTF 2009 policy on the probability of reporting a bi-annual mammogram under the parallel trends assumption: that in the absence of the policy update the bi-annual mammogram trends in the exposed group would have been similar (parallel) to the trends in the unexposed group. We empirically test this assumption by respecifying our primary model in equation 2 as an event-history study:

3) 2) $Y_{iast} = YEAR_t * UPSTF_{at} + STATE_s + YEAR_t + STATE_s * YEAR_t + X'_{ist}$

Here, we no longer exclude the year 2010. We consider the year 2000 to be the reference period for our event-history study. Now, we estimate a set of $USPSTF_{at}$ coefficients interacted with each even year in the study period (2000-2018). If the pre-exposure (2002-2010) coefficient estimates are statistically significantly different than zero, that would cast doubt on our parallel trends assumption and limit the potential internal validity of our Difference-in-Difference estimates. Insignificant pre-exposure estimates provide empirical support for, but cannot confirm our identifying assumption.

© 2025 Semprini J et al. *JAMA Network Open.*
