# Supplementary material for: Socioeconomic and Geographic Differences in Mammography Trends Following the 2009 USPSTF Policy Update
Source: JAMA Netw Open. 2025 Feb 5;8(2):e2458141. doi: 10.1001/jamanetworkopen.2024.58141 (PMC11800017; doi:10.1001/jamanetworkopen.2024.58141)
Supplement: Supplement 2. — Data Sharing Statement [file jamanetwopen-e2458141-s002.pdf]

Data Sharing Statement

Semprini. Socioeconomic and Geographic Differences in Mammography Trends Following the 2009 USPSTF Policy Update. *JAMA Netw Open*. Published February 05, 2025.
doi:10.1001/jamanetworkopen.2024.58141

Data
Data available: No

Additional Information
Explanation for why data not available: Data sharing is limited by third-party restrictions. All analytic code creating, cleaning, and analyzing the data will be publicly available on the corresponding author's repository.
